# Multimodality Imaging of a Ruptured Ovarian Dermoid Cyst Presenting As Disseminated Granulomatous Inflammation: A Radiology Case Report

**DOI:** 10.7759/cureus.96920

**Published:** 2025-11-15

**Authors:** Gearry Mohanraj, Dilrukshi Gunatillake

**Affiliations:** 1 Radiology, Queen Elizabeth Hospital, London, GBR

**Keywords:** chemical peritonitis, dermoid cyst, dermoid cyst rupture, diagnostic pitfall, fdg pet ct, imaging mimic, mature cystic teratoma, mri pelvis, multimodality imaging, radiology

## Abstract

Dermoid cysts develop when epithelium and tissue are enveloped in a sac. A rupture of an ovarian dermoid (mature cystic teratoma) is uncommon and can rarely mimic disseminated malignancy on radiological imaging. This case describes an adult woman developing progressive abdominopelvic fluorodeoxyglucose (FDG)-avid lesions following laparoscopic dermoid cystectomy. Multiple imaging modalities, including magnetic resonance imaging (MRI) and positron emission tomography/computed tomography (PET/CT), were interpreted as possible metastatic ovarian carcinoma. Histology of biopsies following this revealed non-necrotising granulomatous inflammation with no evidence of cancer. The recognition of this benign mimic is important to avoid misdiagnosis and unnecessary stress for patients.

## Introduction

Within ovarian germ cell tumours, teratomas are the commonest subtype, being broadly classified as mature (benign), immature (malignant) and monodermal variants. Dermoid cysts (mature cystic teratomas) are the most common ovarian germ cell tumours and contain well-differentiated tissue, including sebum, hair, keratin elements or calcification, which can form a Rokitansky nodule (dermoid plug) [1,2].

The majority of patients with cysts are asymptomatic with diagnosis made on incidental imaging. However, sometimes symptoms such as abdominal pain are reported [1]. Rupture of these cysts is uncommon, with studies reporting an incidence of less than 1% [2]. When rupture does occur, a leakage of lipid-rich fluid into the peritoneal cavities can provoke intense sterile inflammation known as chemical peritonitis. On imaging, dermoid cysts characteristically demonstrate intralesional fat, calcification and sometimes a Rokitansky nodule [1,2].

However, imaging can be deceptive; for instance, on CT or MRI, omental thickening or free-fluid levels can be interpreted as peritoneal carcinoma [2,3]. Fluorodeoxyglucose-positron emission tomography (FDG-PET) further adds diagnostic complexity as the increased activated macrophages and lymphocytes demonstrate high glucose. Metabolism increases tracer uptake, which makes it difficult to distinguish that of malignancy [4]. Therefore, recognising these rare but important mimics and knowledge of previous imaging aids in preventing misdiagnoses and inappropriate oncological management.

## Case presentation

**Figure 1:**
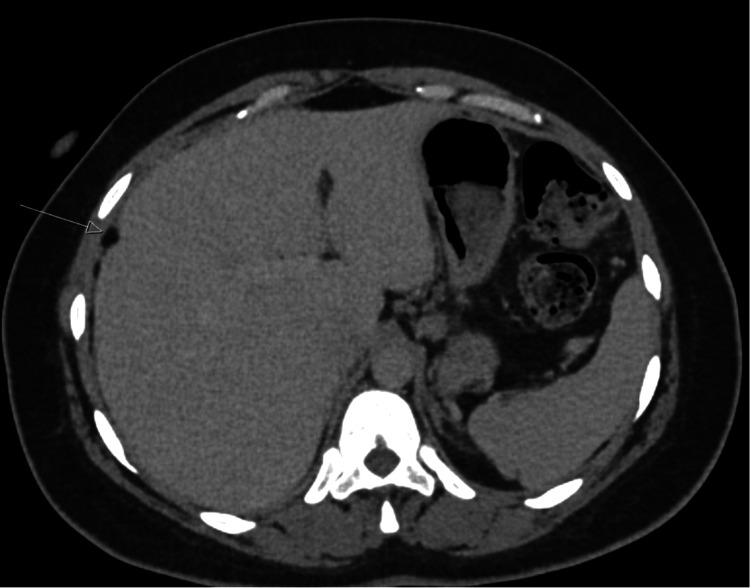
CT Abdomen and Pelvis Demonstrating Fat Spillage Axial CT abdomen showing scattered foci of fat attenuation (arrow) in the pelvis and right subphrenic space, consistent with postoperative dermoid content leakage.

A patient in their late 30s was found incidentally to have asymptomatic bilateral ovarian dermoid cysts during imaging for a retained intrauterine device (IUD). The patient underwent laparoscopic removal of one cyst, while excision of the contralateral lesion was deferred to a later date. This was a surgical decision as the contralateral cyst was smaller than anticipated intraoperatively. Following surgery, the patient developed abdominal pain, low-grade fever and raised inflammatory markers. CT of the abdomen and pelvis revealed scattered foci of fat attenuation within the peritoneal cavity and right subphrenic region, consistent with leakage of dermoid contents or previous rupture (Figure 1).

**Figure 2:**
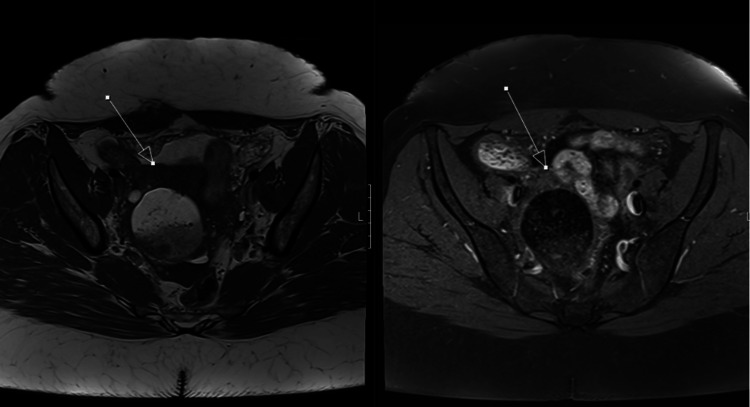
MRI Pelvis Showing Residual Dermoid Cyst and Capsular Discontinuity Axial T1-weighted (left) and T1 fat-saturated (right) images of the pelvis demonstrating a right-sided dermoid cyst with an internal fat-fluid level. Additionally, a fat–fluid level can be visualised within the peritoneal cavity between bowel loops (arrow), consistent with rupture.

MRI of the pelvis shows a residual 7-mm right-sided dermoid cyst with suspicion of fat-fluid levels in the peritoneal cavity and subtle capsular discontinuity (Figure 2). These findings support a diagnosis of focal rupture.

**Figure 3:**
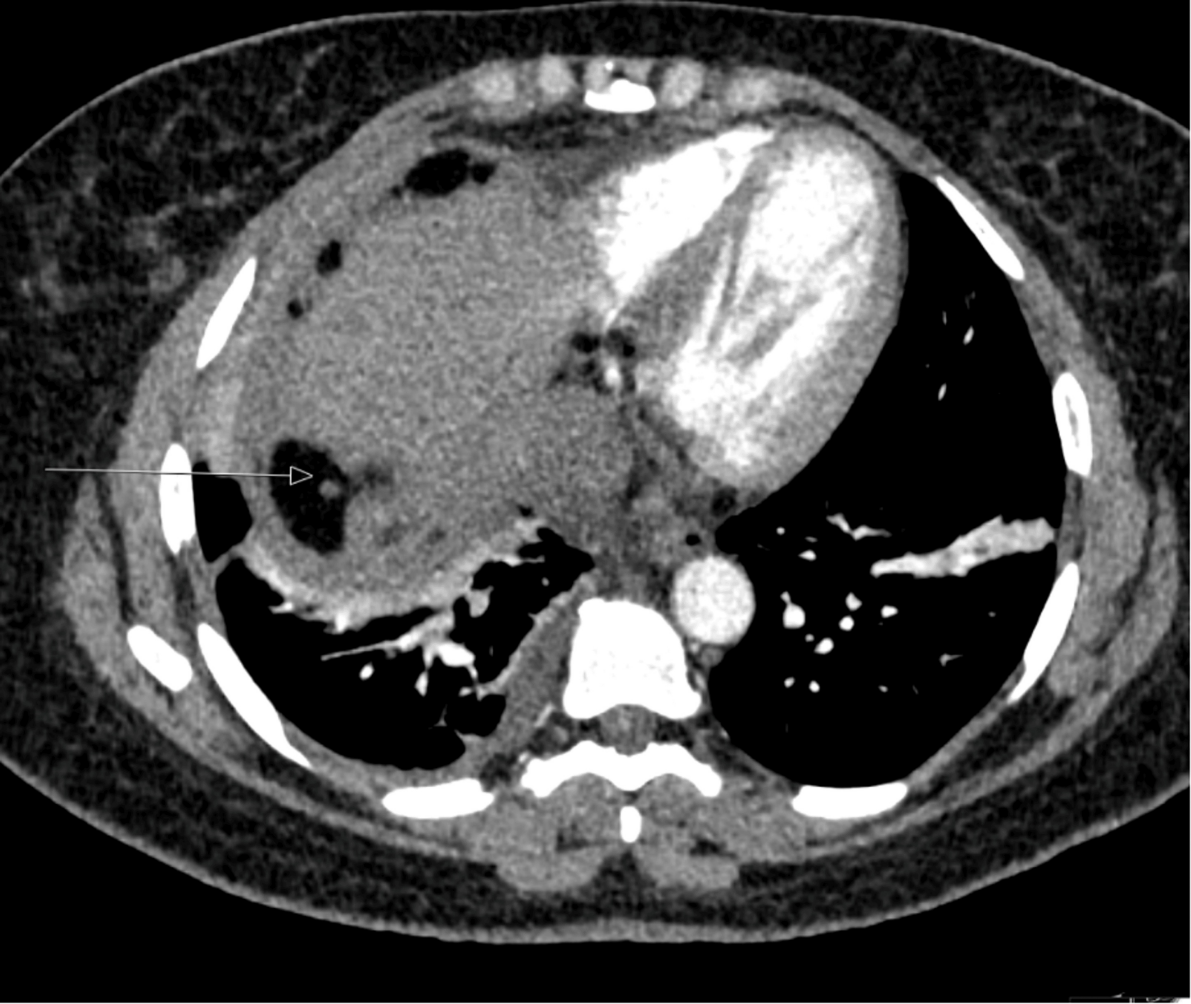
CTPA Showing Right-Sided Consolidation Axial CTPA image showing no pulmonary embolism but right lower-lobe consolidation and adjacent pleural thickening, consistent with inflammatory change and with a significant fat lobule under the diaphragm (arrow). CTPA: computed tomography pulmonary angiogram.

Concurrently, the patient presented for multiple admissions with pleuritic chest pain and dyspnoea. A computed tomography pulmonary angiogram (CTPA) was performed to exclude pulmonary embolism (PE). The study demonstrated no evidence of acute PE but revealed a right-sided mass-like consolidation with adjacent pleural thickening (Figure 3). These findings raised the possibility of postoperative infection or inflammatory changes, and she was commenced on empirical antibiotic therapy. Despite antibiotic therapy, the pulmonary opacity slowly progressed on serial imaging.

**Figure 4:**
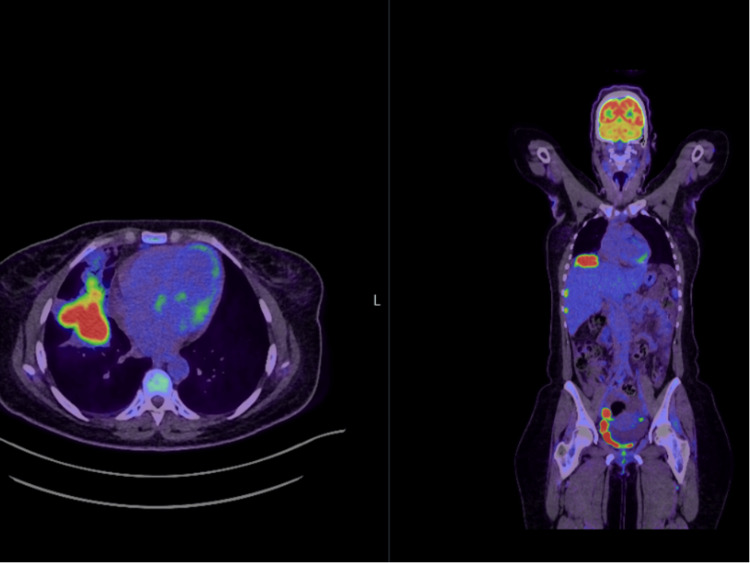
FDG-PET/CT Showing Subphrenic and Pelvic FDG-Avid Lesions Fused FDG-PET/CT image showing intense tracer-avid right pulmonary mass (left: axial view) but on closer inspection located in the subphrenic region (right: coronal view). Additional peritoneal and pelvic foci of uptake are seen, initially suspicious for disseminated malignancy. FDG-PET: fluorodeoxyglucose-positron emission tomography.

To further investigate for malignancy or infection, an FDG-PET/CT was done. The scan demonstrated an intense FDG-avid focus beneath the right diaphragm (subphrenic region) with peritoneal and pelvic deposits. This was initially reported as a right pulmonary mass with pleural, peritoneal and pelvic deposits (Figure 4). However, upon re-review, the uptake was confirmed to be remotely subphrenic with peritoneal and pelvic deposits. Based on the imaging and clinical presentation, the finding was initially interpreted as disseminated malignancy, likely metastatic gynaecological carcinoma. Importantly, a prior history of the ruptured dermoid cyst was not recalled or correlated at the time of the PET/CT interpretation. This led to an overestimation of disease and consideration of oncological management.

**Figure 5:**
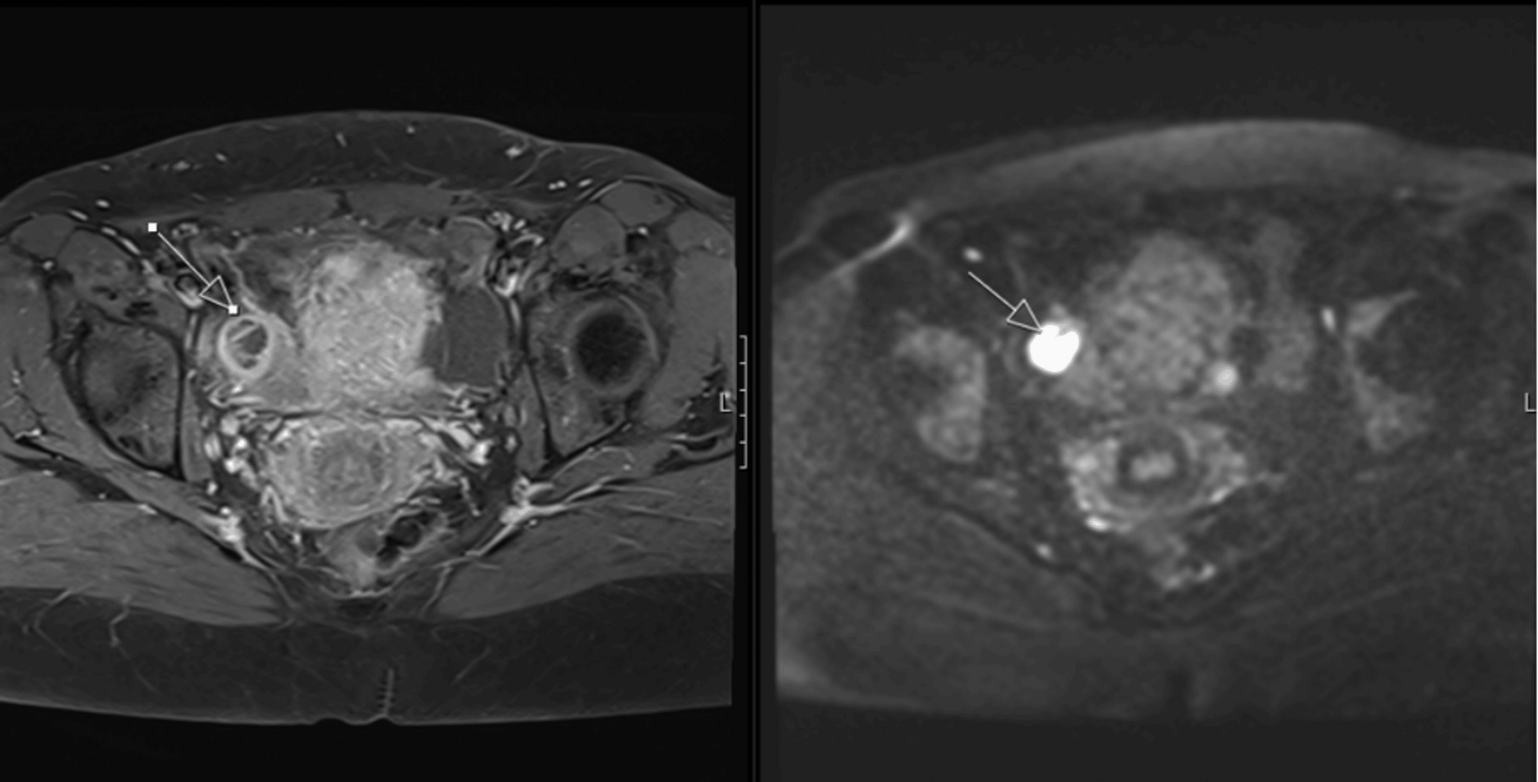
Follow-Up MRI Pelvis Showing Enhancing Adnexal Nodules Post-contrast MRI pelvis demonstrating enhancing soft-tissue nodules in adnexa (arrow) with restricted diffusion, correlating with FDG-avid lesions seen on PET/CT. Post-contrast T1-weighted image (left) and diffusion-weighted image (right). FDG: fluorodeoxyglucose; PET: positron emission tomography.

An interval MRI pelvis was performed for further characterisation, which demonstrated T2 intermediate-signal soft-tissue nodular areas within both adnexal regions, which were enhanced on post-contrast imaging and showed areas of restricted diffusion (Figure 5). These features appeared suggestive of neoplastic disease. The nodular foci corresponded to areas of increased uptake on the recent PET/CT. Compared with the previous MRI that was taken several months earlier, the mass adjacent to the bladder on the right had increased in size, and abnormal soft-tissue nodularity in the left adnexal region was also more significant. The differentials included primary ovarian malignancy or metastatic disease.

CT-guided biopsy of the subdiaphragmatic lesion demonstrated non-necrotising granulomatous inflammation with no malignant cells, as per histology, and ascitic fluid cytology was negative. During multidisciplinary review, correlation with earlier imaging revealed the history of dermoid rupture and intra-abdominal fat spillage, thus providing a unifying benign explanation for the disseminated FDG-avid process. The image appearances, therefore, were reclassified as granulomatous inflammation secondary to dermoid rupture and chemical peritonitis.

The patient is being managed conservatively with supportive care. No further surgical intervention has been required. Seven months following the initial surgery, the patient remains clinically stable and ongoing monitoring has been planned to assess for the gradual resolution of inflammatory changes.

## Discussion

Pathophysiology

The rupture of dermoid cysts releases sebaceous material, keratin and hair into the peritoneal cavity. The components act as potent irritants, creating a chronic granulomatous foreign body reaction composed of lipid-laden macrophages and giant cells [2,3]. Droplets of fat can migrate through the diaphragmatic lymphatic channels or venous routes to the thoracic cavity, leading to pleural granulomatous lesions or fat emboli [3].

Imaging features and diagnostic pitfalls

CT findings included the free intraperitoneal fat, fat-fluid levels and peritoneal thickening. MRI is useful in confirming lipid content and demonstrating subtle capsular defects [1,2]. FDG-PET/CT frequently demonstrates high tracer uptake in inflammatory deposits. Therefore, the distribution of uptake may be similar to that of metastases, but the presence of fat densities and clinical context should increase suspicion of a benign inflammatory cause [4].

This case illustrates the risk of not reviewing previous imaging. The earlier CT and MRI findings, which demonstrate fat spillage and capsular rupture, were available but not correlated initially when the PET/CT was interpreted. Without this context, the widespread FDG-avid lesions were misattributed to malignancy, leading to overestimation and consideration of oncological management. Therefore, correlation with prior imaging is critical to prevent misdiagnosis, especially in complex postoperative cases. The imaging differentials can encompass metastatic ovarian carcinoma and tuberculous peritonitis [4,5]. Absence of malignant cells on biopsy and characteristic fatty features increases the likely differential of post-rupture chemical peritonitis [5].

Comparison with the literature

Several authors have also described cases where widespread FDG-avid lesions mimic various malignancies [6]. The time interval between surgery and presentation may vary from days to months. In reports, conservative management has led to spontaneous improvement once the correct diagnosis has been made, and surgery is indicated if cysts are greater than 5 cm [5].

Learning points

This case highlights that rupture of an ovarian dermoid cyst can result in disseminated granulomatous inflammation, which can mimic malignancy on imaging. Recognising and being aware of this can avoid misinterpretation of radiological findings. Secondly, a thorough review of previous images and awareness of the patient’s history is vital for providing context, leading to an accurate diagnosis. It is also crucial to recognise that inflammatory lesions can demonstrate high tracer uptake on FDG-PET/CT findings, which is often hard to distinguish from malignancy. Finally, it is important to seek histopathological biopsy and multidisciplinary discussions to aid in confirmatory diagnosis.

## Conclusions

The rupture of ovarian dermoid cysts can produce widespread granulomatous inflammation that closely mimics disseminated malignancy on multimodality imaging, particularly on FDG-PET/CT. This case demonstrates the significance of correlating findings with previous imaging and history to reduce misdiagnosis. Through awareness of this benign mimic, unnecessary oncological treatment, invasive procedures and emotional distress for the patient can be prevented. Finally, multidisciplinary discussions and biopsy confirmation remain essential to ensure an accurate diagnosis.
